# Interplay between acute phase response and coagulation/fibrinolysis in chronic spontaneous urticaria

**DOI:** 10.1186/s13223-018-0255-8

**Published:** 2018-07-18

**Authors:** R. Grzanka, A. Damasiewicz-Bodzek, A. Kasperska-Zajac

**Affiliations:** 1 Department of Internal Diseases, Dermatology and Allergology, SMDZ in Zabrze, Medical University of Silesia in Katowice, Katowice, Poland; 2 Department of Chemistry, SMDZ in Zabrze, Medical University of Silesia in Katowice, Katowice, Poland; 3 European Center for Diagnosis and Treatment of Urticaria, Zabrze, Poland; 4 Department of Internal Diseases, Dermatology and Allergology, ul. M. Curie-Skłodowskiej 10, 41-800 Zabrze, Poland

**Keywords:** D-dimer, CRP, Inflammation, Chronic spontaneous urticaria, Coagulation/fibrinolysis, Acute phase response

## Abstract

**Background:**

Chronic spontaneous urticaria (CSU) is associated with activation of systemic inflammatory response and coagulation/fibrinolysis.

**Aim:**

To study whether there is a relationship between the acute phase response and coagulation/fibrinolysis in chronic spontaneous urticaria (CSU) patients.

**Methods:**

Serum concentrations of C-reactive protein (CRP) and interleukin 6 (IL-6), key markers of acute phase response and of D-dimer, a marker of fibrin turnover were investigated in 58 CSU patients assessed with the urticaria activity score (UAS) and the controls.

**Results:**

Serum concentrations of IL-6, CRP, and D-dimer were significantly higher in CSU patients as compared with the controls. We found statistically significant correlations between D-dimers concentrations and the inflammatory markers: CRP and IL-6 as well as UAS.

**Conclusions:**

Markers of inflammation (IL-6 and CRP) and of fibrinolysis (D-dimer) are related to each other in CSU, suggesting a possible cross-talk between inflammation and coagulation/fibrinolysis. It might be implicated in pathogenesis of the disease and may be associated with higher risks of cardiovascular diseases in CSU patients.

## Background

Chronic spontaneous urticaria (CSU) is associated with activation of systemic inflammatory response and coagulation/fibrinolysis [1–3].

C-reactive protein (CRP) and interleukin 6 (IL-6) are markers of acute phase response (APR) and underlying systemic inflammation, while D-dimer is a marker of fibrin turnover and fibrinolysis. In general, elevated CRP, IL-6 and D-dimer concentrations are not specific responses, which may increase during many inflammatory diseases [4–7].

Previous studies have reported that circulating IL-6, CRP and D-dimer were elevated in CSU patients, relating to severity/activity of the disease [1, 8–10].

Associations between activation of coagulation and fibrinolytic systems and the inflammatory response are found in pathogenesis of multiple diseases [4–7], yet little is known about the cross-talk between inflammation and thrombotic/fibrinolytic pathways in CSU.

Given these facts, we sought to see whether there was any correlation between the markers of the acute phase response and coagulation/fibrinolysis in CSU. Therefore, we measured serum concentrations of CRP, IL-6 and D-dimer as well as their relationships in patients with CSU and the healthy subjects.

## Methods

We enrolled 58 selected patients with active CSU of unknown causes and without any concomitant diseases, including chronic inducible urticaria (CIndU) to obtain homogeneous group; (the median of age was 39 years, range 21–45 years; 17 males and 41 females) and 22 matched for age, sex healthy controls, the hospital staff. The mean disease duration was 30.5 months (range 7–62 months). All patients fulfilled the EAACI/GA(2)LEN/EDF/WAO criteria for CSU (spontaneous appearance of wheals/no obvious external specific trigger daily, or almost daily for ≥ 6 weeks) [11]. Throughout the UAS evaluation period, none of the patients showed any manifestations of angioedema during 4 days (i.e. before blood tests). 47% of CSU patients had a positive history of angioedema.

CIndUs were excluded based on the patient history and the results of provocation testing, including FricTest^R^, Temptest^R^, dermographometer according to recommendations [12].

The disease activity in CSU patients was measured by the number of wheals/24 h [(0) no, (1) mild (< 20), (2) moderate (20–50), (3) (> 50 wheals or large areas)], and the patient’s assessment of itch using a four-point scale [(0) no itch, (1) mild, (2) moderate, (3) severe/intensive)] [11]. The urticaria activity score (UAS) was calculated during 4 days, modified UAS7 as described  [2]. Patients were classified into two groups; 31 with mild CSU (0–8 points) and, 27 with moderate-severe CSU (9–24 points).

A panel of tests was performed (wider than in everyday practice and recommended in the guidelines, which suggest only differential blood count and CRP and/or ESR as basic test with no further measures but those based on the patient history and examination) [11], to select a homogeneous group and to exclude concomitant diseases/conditions that may lead to an increase in systemic inflammatory response markers.

Each patient underwent detailed clinical examination; routine biochemical investigations including hematologic, renal, thyroid and hepatic function tests; rheumatoid factor (RF), immunoglobulins G, E, M (IgG, IgE and IgM), serum protein electrophoresis and complement components (C3 and C4) analysis; anti-cyclic citrullinated peptide autoantibodies (anti-CCP), allergen-specific IgE antibodies (to environmental allergens), anti-nuclear antibody (ANA), anti-TPO, antibodies specific to the *B. burgdorferi* antigens (Western Blot), anti-*Ascaris lumbricoides* and anti-*H. pylori* antibodies, toxoplasma and toxocariasis specific antibodies, anti-neutrophil cytoplasmic antibodies assays; serum lipid profile; stool ova, parasites and *H. pylori* antigen as well as routine urine exam; dental and chest X-rays; abdominal and thyroid ultrasound; some other investigation depending on medical history e.g. computed tomography (CT) of the sinuses, gastroscopy and colonoscopy procedures; anti-endomysial, anti-transglutaminase and antiphospholipid antibodies assays.

All patients underwent stomatological, gynaecological, and laryngological examinations. The exclusion criteria were as follows: infectious atopic and inflammatory disease, rheumatoid or other autoimmune diseases, known malignancy, hepatic, thyroid or renal dysfunction, diabetes mellitus, cardiovascular diseases, immunoglobulin disorders and obesity.

All patients were free of second generation H1-antihistamine administration within the last 4 days, and glucocorticoids and cyclosporine therapy had been withdrawn at least 8 weeks before. None of these patients were previously treated with omalizumab.

The Ethical Committee of the Medical University of Silesia approved of the study protocol and written consent was obtained from CSU patients and the controls.

### Blood collection

For all measurements, venous blood was collected from the antecubital vein in the morning after an overnight fast. Sera obtained by centrifugation were stored at − 85 °C until analysis of concentrations of CRP, IL-6 and D-dimer. The remaining samples were collected in tubes with different anticoagulant solutions depending on the type of analysis and evaluations were made as part of a standard battery of routine laboratory examinations in CSU patients.

### Assay of CRP, D-dimer and Il-6

Concentrations of C-reactive protein (CRP), D-dimer and interleukin-6 (Il-6) in serum samples were measured by an enzyme-linked immunosorbent assay (ELISA) using commercially available kits: Quantikine ELISA Human C-Reactive Protein/CRP kit by R&D Systems (MN, USA) for CRP, Human D-Dimer SimpleStep ELISA kit ab196269 by Abcam (Cambridge, UK) for D-dimer and Quantikine ELISA Human Il-6 kit by R&D Systems (MN, USA) for Il-6. All assays were performed according to manufacturer’s detailed instructions. The coefficients of variance for intra-assay and inter-assay were for all assays below 8 and 10%, respectively.

### Statistical analysis

The data were presented as the median and interquartile range (IQR). Normal distribution of data was calculated using Shapiro–Wilk’s test. Independent samples between the groups of CSU patients and the controls were compared using non-parametric the *U* Mann–Whitney test. Comparisons between different groups were carried out by the ANOVA rang Kruskal–Wallis test. The correlations (*R* values) were assessed using Spearman’s rank test.

The *p* value of less than 0.05 was considered as statistically significant. All statistical calculations were preformed using STATISTICA for Microsoft Windows 10.0 (StatSoft Poland).

## Results

### Serum concentrations of IL-6, CRP and D-dimer

**Fig. 1:**
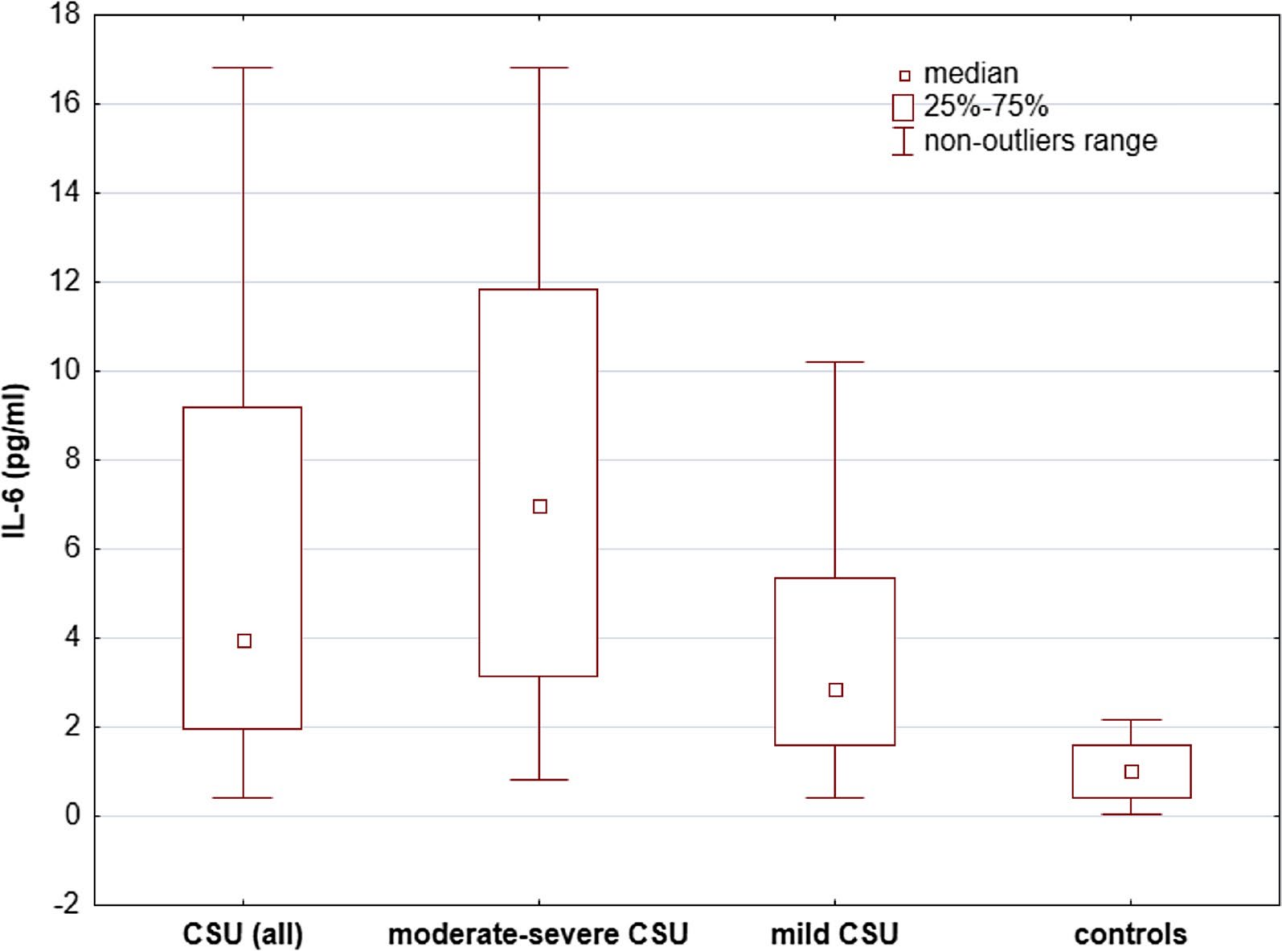
Serum IL-6 concentrations in CSU patients grouped by activity/severity of the disease and the controls. There were significantly higher concentrations of IL-6 in moderate-severe and mild CSU patients versus the controls (< 0.0001)

**Fig. 2:**
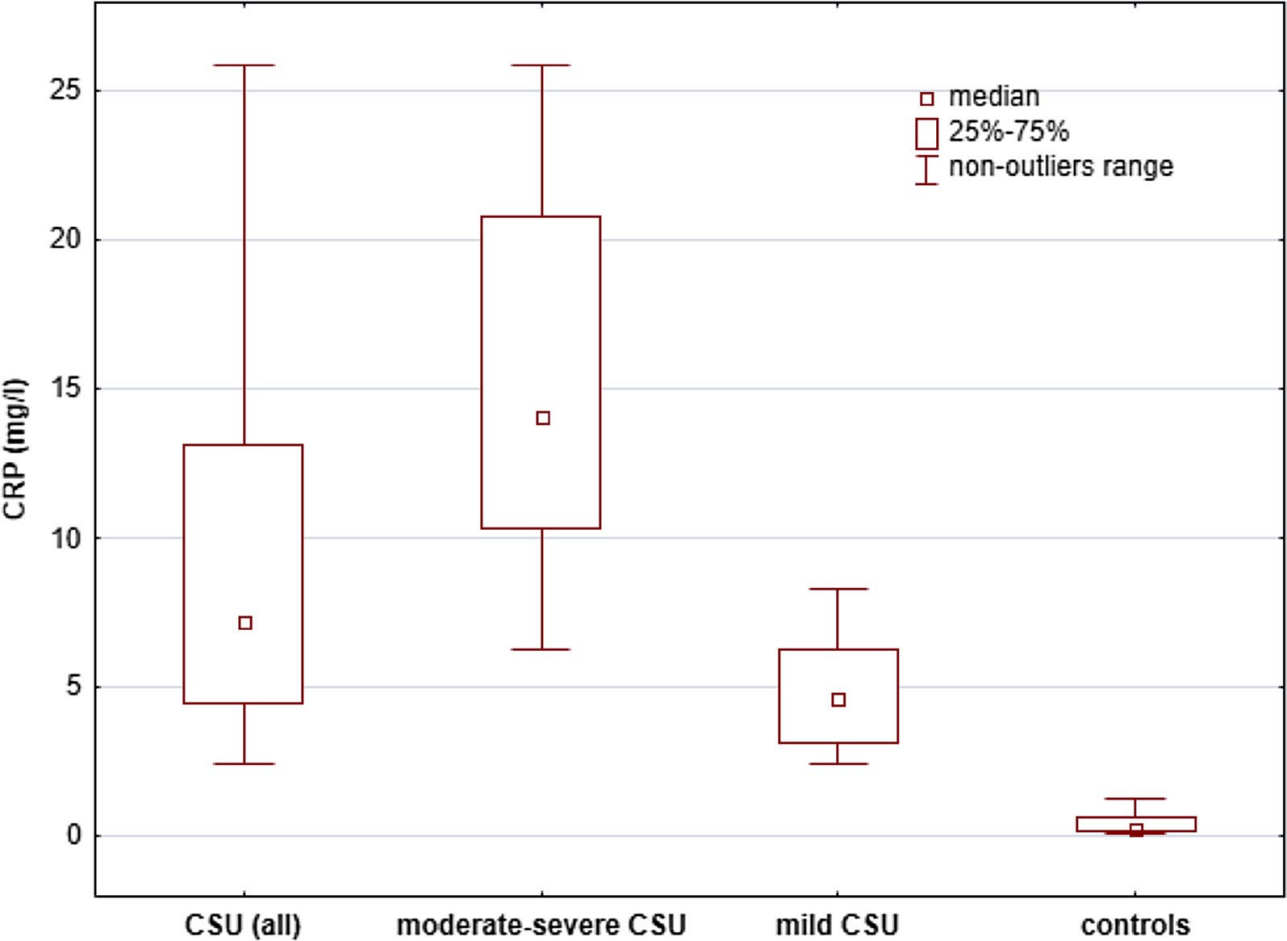
Serum CRP concentrations in CSU patients with different activity/severity of the disease and the controls. There were significantly higher CRP concentrations in moderate-severe and mild CSU patients versus the controls (p < 0.0001)

**Fig. 3:**
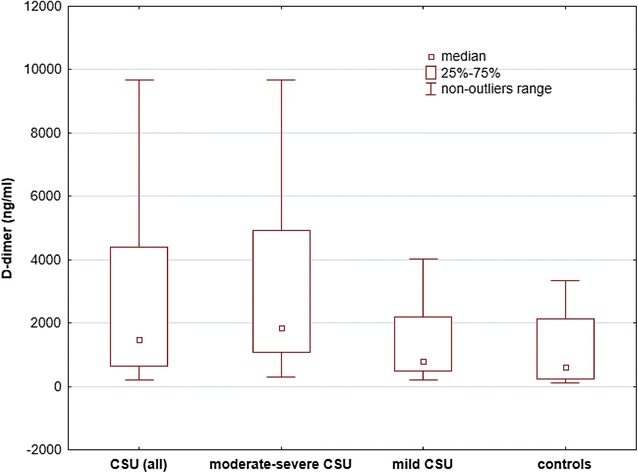
Serum D-dimer concentrations in CSU patients grouped by activity/severity of the disease and the controls. The concentrations were significantly higher in moderate-severe CSU and CSU (all patients) as compared with the controls (p < 0.001 and p < 0.01, respectively). There were no significant differences between mild CSU and the controls (p > 0.05)

The CSU patients showed significantly higher concentrations of IL-6 [median and IQR: 3.95 (1.98–9.2) vs 1.0 (0.43–1.58) pg/ml; *p* < 0.0001), CRP [7.26 (4.46–13.14) vs 0.3 (0.21–0.65) mg/l; *p* < 0.0001) and D-dimer [median and range: 1475.32 (624.62–4406.19) vs 598.75 (225.15–2139.73) ng/ml; p < 0.001] than the controls (Figs. 1, 2, 3).

The moderate-severe CSU patients had significantly higher concentrations of IL-6 [median and IQR: 6.96 (3.16–11.86) vs 2.86 (1.58–5.35) vs 1.0 (0.43–1.58) pg/ml; p < 0.05 and p < 0.0001, respectively], CRP [6.28 (10.37–20.79) vs 4.62 (3.16–6.32) vs 0.3 (0.21–0.65) mg/l; p < 0.001 and *p* < 0.0001, respectively] and D-dimer [median and IQR: 1854.1 (1063.43–4936.06) vs 783.1 (496.29–2195.62) vs 598.75 (225.15–2139.73) ng/ml; p < 0.05 and p < 0.01, respectively] than mild CSU and the controls (Figs. 1, 2, 3).

The mild CSU patients showed significantly higher concentrations of IL-6 [median and IQR: 2.86 (1.58–5.35) vs 1.0 (0.43–1.58) pg/ml; p < 0.001], CRP [4.62 (3.16–6.32) vs 0.3 (0.21–0.65) mg/l; *p* < 0.001] than the controls. In mild CSU patients D-dimer concentrations did not differ significantly as compared with the controls [median and IQR: 783.1 (496.29–2195.62) vs 598.75 (225.15–2139.73) ng/ml; p > 0.05; Fig. 1, 2, 3].

### Correlations

**Fig. 4:**
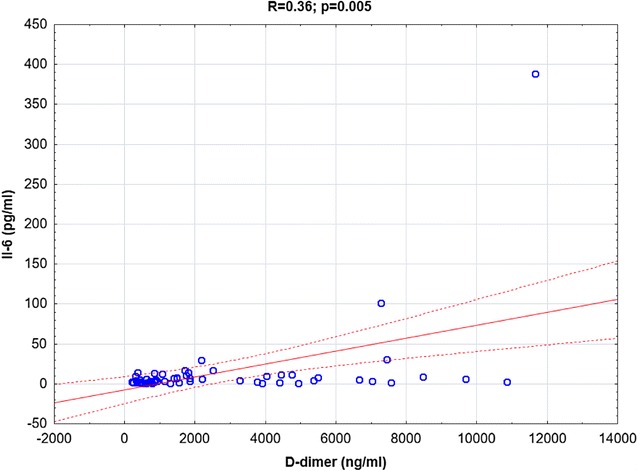
The correlation between IL-6 and D-dimer concentrations in CSU patients

**Fig. 5:**
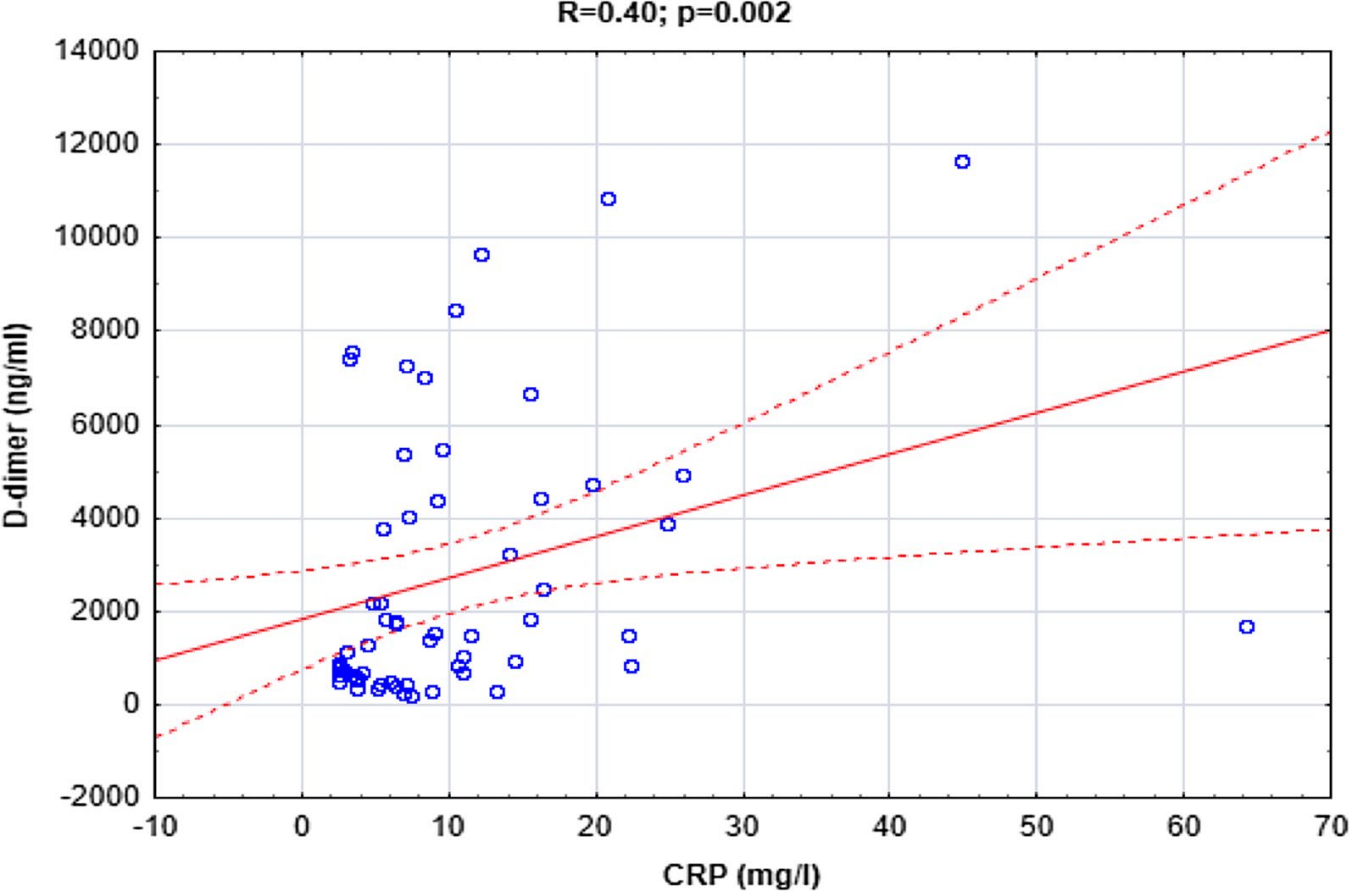
The correlation between CRP and D-dimer concentrations in CSU patients

**Fig. 6:**
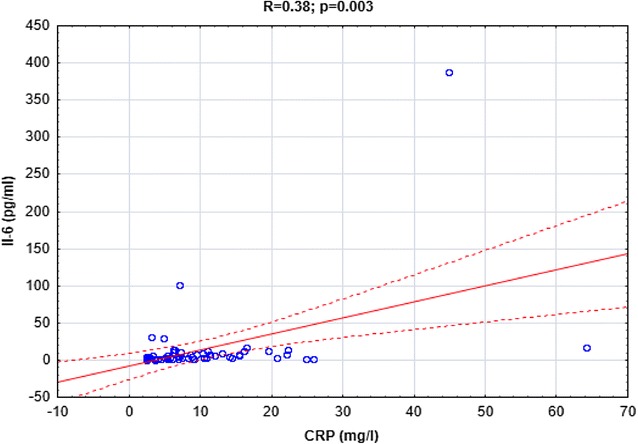
The correlation between IL-6 and CRP in CSU patients

Both IL-6 and CRP were positively correlated with D-dimer (*R *= 0.36, *p *= 0.005 and *R *= 0.4, *p *= 0.002, respectively, Figs. 4, 5.) In addition there were significant correlations between IL-6 and CRP concentrations (*R *= 0.38, *p *= 0.003, Fig. 6).

There were significant correlations between UAS and IL-6, CRP, D-dimer (R = 0.4, p = 0.0019; R = 0.98, p = 0.00000; R = 0.39, p = 0.002).

## Discussion

It is known, that chronic low-grade inflammation and activation of coagulation/fibrinolytic systems are present in CSU, especially in patients with more severe disease activity [3, 13]. The present findings are consistent with previous studies, indicating that CSU is characterized by the presence of elevated circulating markers of acute phase response (IL-6 and CRP) and of fibrin turnover (D-dimer) [1, 8–10]. Kasperska-Zajac et al. observed significant correlation between IL-6 and CRP and the severity/activity of CSU [1, 8]. Asero et al. found that D-dimer concentrations were higher in CSU patients, suggesting activation of coagulation/fibrinolytic systems in patients with more severe disease activity [9, 10]. Based on large study it has been indicated that the assessment of CRP may help to optimize the management of CSU patients [14]. Kolkhir et al. reported significant association between CRP and IL-6, D-dimer, which was confirmed in our study [14]. In addition, we describe significant association between IL-6 and D-dimer (p = 0.005). Interestingly, a significant correlation was observed between CRP and ERS, leukocytes in CSU [14]. We and other found a positive correlation between these markers, likely reflecting the potential interplay between coagulation/fibrinolysis and acute phase response in CSU.

Therefore, we suggest that the underlying mechanism for this higher activity of coagulation/fibrinolysis system may result from local and/or systemic inflammatory response in CSU.

Activated local and systemic inflammation leads to an activation of the hemostasis system, which vice versa may modulate the immune-inflammatory response by various mechanisms [4–7].

The key factor linking coagulation and inflammation is the tissue factor (TF). Its expression is directly or indirectly induced by multiple proinflammatory cytokines (IL-6, tumor necrosis factor (TNF)-α, IL-1 [4, 5, 15] and CRP [16], which all are increased in CSU [13, 17, 18]. It has been suggested that IL-6 may directly stimulate coagulation in severe inflammation [19]. The recombinant human IL-6 was able to induce activation of coagulation as reflected by release of thrombin-antithrombin III complexes and in the prothrombin activation fragment F1 + 2 [19]. Up-regulation of TF leads to activation of the extrinsic coagulation pathway with subsequent fibrinolysis and release of the cross linked fibrin degradation products, including D-dimer. These components of the fibrin pathway may also modulate the inflammatory response [4, 5, 15]. It has been indicated that local fibrinolysis in inflammatory lesions is associated with the release fibrinogen-derived lymphocyte-suppressive peptides providing an immunosuppressive mechanism for local cellular immune response [20].

Beyond the major screening role in the diagnosis of venous thromboembolism and pulmonary embolism, increased D-dimer concentration was found to be associated with a number of processes, including inflammation and infection [5–7]. Interestingly, it has been reported that D-dimer induces the production of biologically active IL-1 beta, IL-6 from human monocytes [21].

Several markers, especially acute phase reactants, have been suggested to provide information about the CSU severity. In particular, IL-6, a key regulator of CRP synthesis was strongly associated with the clinical symptoms in CSU [1, 8].

In the present study, markers of acute phase response (IL-6 and CRP) and of fibrin turnover (D-dimer) were associated with each other in CSU patients. These findings may relate to the association of inflammation and fibrin turnover in urticarial lesions. It has been indicated that CSU is characterized by activation of the extrinsic pathway of clotting cascade, probably due to TF expression by activated eosinophils in the urticarial lesions [22, 23]. Interestingly, TF reactivity was observed in all skin specimens from chronic urticaria patients [22, 24]. Local inflammatory responses in the urticarial lesions appear to promote fibrin formation and fibrinolysis, resulting in elevated D-dimer concentration [3, 22, 24].

It seems that local fibrin formation and lysis, resulting in production of fibrin degradation products, including D-dimer are part of the immune-inflammatory response in CSU and the interplay between inflammatory and coagulation/fibrinolysis factors in CSU may lead to maintenance/amplification of urticarial inflammation.

D-dimer is a sensitive marker of fibrinolytic activity, which may occur both, intravascularly and extravascularly. It has been indicated that there is a predominant extravascular location of fibrin formation in urticarial lesions [3, 24].

Considering the fact that low-grade systemic inflammatory state, with an increase in the concentration of CRP as a marker is a risk factor for cardiovascular diseases [6]. In the future, one should answer the question whether a similar situation does not occur in CSU patients with uncontrolled symptoms accompanied by systemic inflammatory response. Activated inflammatory and coagulation/fibrinolysis response contribute to increased risk of cardiovascular events [6, 7]. Therefore, it seems that CSU patients should be intensively treated to ensure comprehensive control of their symptoms and to reduce both processes.

Nevertheless, our study has some limitations. The study allowed for recognition of the association, but we cannot prove the existence of any causal relationship. It seems important to perform repeated blood analyses after symptomatic treatment and CSU remission. Sequential assessments of D-dimer and CRP concentrations, related to severity/activity of CSU might provide stronger evidence of a causal relationship between coagulation/fibrinolysis activation and inflammation. Therefore, more studies are required to verify such hypothesis.

A growing body of research points to the association between these markers and the severity/activity of CSU [14]. At present there are no better tests to evaluate CSU patients than UAS7 and urticaria control test (UCT) [11]. Analysis of CRP and D-dimer is rapid and inexpensive, therefore combining measurement of both CRP and D-dimer may be a logical and practical tool to improve monitoring of CSU activity/severity in patients with low compliance. Dynamic measurement of CRP and D-dimer concentrations may be clinically useful for evaluation of the treatment effectiveness or the disease exacerbation, especially in CSU patients with low compliance with UAS monitoring. In addition, despite the absence of clinical symptoms of the disease, elevated CRP and D-dimer may suggest persisting subclinical urticarial inflammation and indicate the need for more intensive therapy. CRP and D-dimer concentrations decline during reduction or disappearance of CSU symptoms following anti-histamine or omalizumab therapy and increase during exacerbation (not published observation). In addition, adequate CRP and D-dimer interpretation is important due to the frequent usage of the tests in the context of concurrent infectious/inflammation or hypercoagulation processes, which can independently occur in CSU patients. In some CSU patients with coexisting inflammatory processes (e.g. tonsillitis, sinusitis) CRP may be increased, but not D-dimer (unpublished observation). Most importantly, simultaneous measurement of both markers seems to be more effective in differential diagnosis. Allowing for identification of some patients with individual contribution of co-infections/inflammation or co-morbidities to variance in CRP concentration. Taking into account the known inhibitory effect of corticosteroids on IL-6 production, a key factor in CRP production [25], D-dimer seems to be better marker of CSU activity/severity in CSU patients treated with corticosteroids due to exacerbation.

However, any recommendations and potential clinical use should be preceded by more extensive studies.

In conclusion, markers of acute phase response (IL-6 and CRP) and of fibrin turnover (D-dimer) are related to each other in CSU patients, suggesting the possible interaction between these markers in the disease. There may be an interplay between inflammation and coagulation/fibrinolysis pathways in the pathogenesis of CSU.

Clinical usefulness of these marker, especially D-dimer for determining the severity/activity of CSU, is not completely clear.
